# Investigating Intergenerational Differences in Human PCB Exposure due to Variable Emissions and Reproductive Behaviors

**DOI:** 10.1289/ehp.1002415

**Published:** 2010-12-14

**Authors:** Cristina L. Quinn, Frank Wania, Gertje Czub, Knut Breivik

**Affiliations:** 1 Department of Chemistry and Department of Physical and Environmental Sciences, University of Toronto Scarborough, Scarborough, Ontario, Canada; 2 Swedish Chemicals Agency, Sundbyberg, Sweden; 3 Norwegian Institute for Air Research, Kjeller, Norway; 4 University of Oslo, Department of Chemistry, Oslo, Norway

**Keywords:** environmental fate, human exposure, reproductive characteristics, modeling organic contaminants, PCBs, time-variant emissions

## Abstract

**Background:**

Reproductive behaviors—such as age of childbearing, parity, and breast-feeding prevalence—have changed over the same historical time period as emissions of polychlorinated biphenyls (PCB) and may produce intergenerational differences in human PCB exposure.

**Objectives:**

Our goal in this study was to estimate prenatal, postnatal, and lifetime PCB exposures for women at different ages according to year of birth, and to evaluate the impact of reproductive characteristics on intergenerational differences in exposure.

**Methods:**

We used the time-variant mechanistic model CoZMoMAN to calculate human bioaccumulation of PCBs, assuming both hypothetical constant and realistic time-variant emissions.

**Results:**

Although exposure primarily depends on when an individual was born relative to the emission history of PCBs, reproductive behaviors can have a significant impact. Our model suggests that a mother’s reproductive history has a greater influence on the prenatal and postnatal exposures of her children than it does on her own cumulative lifetime exposure. In particular, a child’s birth order appears to have a strong influence on their prenatal exposure, whereas postnatal exposure is determined by the type of milk (formula or breast milk) fed to the infant.

**Conclusions:**

Prenatal PCB exposure appears to be delayed relative to the time of PCB emissions, particularly among those born after the PCB production phaseout. Consequently, the health repercussions of environmental PCBs can be expected to persist for several decades, despite bans on their production for > 40 years.

Polychlorinated biphenyls (PCBs) are a group of anthropogenic persistent organic pollutants that were used in a variety of applications until production was banned beginning in the 1970s (Safe 1994). Human exposure to PCBs typically occurs through consumption of contaminated food (Moser and McLachlan 2002). One of the first incidents to indicate the toxicity of PCBs occurred in 1968 when > 1,000 people in Japan became ill after ingesting PCB-contaminated rice oil (Fujiwara 1975). A similar incident occurred in Taiwan in 1979 (Chen et al. 1980). The populations affected by these incidents, referred to as Yusho and Yucheng, respectively, have been well studied and serve as references for the hazardous effects of high PCB exposure (Toft et al. 2004). Yusho adults exposed to PCBs suffered from dermal abnormalities and neurologic disorders (Aoki 2001), and Yusho infants exposed to PCBs *in utero* exhibited, among other symptoms, decreased motor skills, growth impairment, and reduced IQ (Aoki 2001; Jacobson et al. 1990). A common symptom of both adult and *in utero* exposure was reduced fecundity (Guo et al. 2000; Hsu et al. 2003) and abnormal reproductive development (Toft et al. 2004, 2006).

Three different time periods of PCB exposure are considered in epidemiologic studies: prenatal exposure, postnatal exposure, and lifetime exposure (Aoki 2001; Jacobson et al. 1990). Prenatal exposure, due to transplacental transfer of PCBs from the mother to the fetus, is usually estimated based on PCB concentrations measured in umbilical cord serum (Jacobson et al. 1990). Postnatal exposure occurs during breast-feeding when maternal milk is contaminated by lipid-soluble PCBs that have accumulated in the mother (Jacobson et al. 1990). Lifetime exposure is the cumulative exposure to a given contaminant over the lifetime of the individual resulting from prenatal, postnatal, childhood, and adult exposure (Alcock et al. 2000). Because emissions of PCBs have varied greatly over time, lifetime exposure depends on the year of birth (Alcock et al. 2000; Moser and McLachlan 2002; Ritter et al. 2009), therefore resulting in intergenerational differences in exposure (IGDE).

Changes in reproductive behaviors during the time period of PCB emissions, including changes in average numbers of children (parity), maternal age at birth, and breast-feeding versus not breast-feeding, may further enhance or reduce IGDE. Body burden variability within a population has been attributed to age (Bergonzi et al. 2009; Dewailly et al. 1996), and declines in body burden in mothers have been attributed to the cumulative duration of breast-feeding (Dewailly et al. 1996; Tajimi et al. 2004). Parity has been both implicated (Vaz et al. 1993; Wang et al. 2009) and disputed (Bergonzi et al. 2009) as a cause of body burden decline.

Using a time-variant model, Alcock et al. (2000) estimated that the body lipid PCB concentration of a woman born in 1950 would decrease by 25% after 6 months of breast-feeding. In the present study, our objectives were to quantitatively determine the extent of prenatal, postnatal, and lifetime exposure relative to atmospheric emissions assuming both hypothetical constant and historical time-variant emissions, and to evaluate the impact of reproductive characteristics on IGDE. To accomplish this, we used the CoZMoMAN model (Breivik et al. 2010) to predict PCB concentrations according to a mechanistic model of their environmental fate and food chain bioaccumulation. Our results identify generations of the population with the greatest possibility of exposure to PCBs and suggest the extent to which reproductive behaviors contribute to IGDE.

## Materials and Methods

All simulations were performed with CoZMoMAN (Breivik et al. 2010), a time-variant multimedia mechanistic model that was created by linking the CoZMo-POP2 model (Wania et al. 2006), which describes contaminant environmental fate, and the ACC-HUMAN model (Czub and McLachlan 2004), which describes human food chain bioaccumulation. CoZMoMAN has been used to simulate time trends in PCB concentrations in air, seawater, soils, sediment, herring, cod, beef, and human breast milk based on historical time-variant PCB emissions for the western Baltic Sea region, which were compared with reported measured values (Breivik et al. 2010). Using the same parameterization as in that study, we estimated time trends for lipid-normalized PCB concentrations in Swedish women born at different times and explored the impact of reproductive behaviors that have undergone changes (specifically, the number of children born to a mother, maternal age at childbirth, and breast-feeding or formula feeding) during the historical PCB emissions time period, first assuming constant emission and dietary intake scenarios, and then assuming time-variant PCB emissions based on historical data.

In this article we focus on model estimates for PCB-153 (2,2′,4,4′,5,5′-hexachlorobiphenyl) according to a single PCB emission scenario that was previously judged to be most realistic and that produced estimates that were most consistent with the observed time trends (Breivik et al. 2010). Specifically, we assumed that 5% of annual imports during the time of PCB production and import were emitted to the atmosphere and, for models of constant emissions, assumed emissions of 0.198 metric tons/year, the average rate of the time-variant emissions for PCB-153 from 1930 through 2100 according to this scenario. In addition, we assumed that the metabolic biotransformation rate of the PCBs was constant over time regardless of body burden or age (Czub and McLachlan 2004), and we used default settings for all other model parameters (Breivik et al. 2010) [for details, including dietary and physiochemical parameters, see Supplemental Material, text and Table 1 (doi:10.1289/ehp.1002415)]. Calculations also were performed for six additional PCB congeners (28, 53, 101, 118, and 180) and for an alternative scenario of historical emission time trends, as described in the Supplemental Material.

The amount of prenatal transfer of each PCB congener was determined by assuming that the infant is in chemical equilibrium (equifugacity) with the mother at the time of birth. The amount of lactational transfer was estimated assuming that breast milk is in chemical equilibrium with the mother’s body tissues and blood. We also assumed that breast-feeding occurs for 6 months, after which the infant receives a regular diet adjusted for age (Moser and McLachlan 2002). Estimates are presented for a default female who is defined as a woman who was breast-fed for 6 months and was the only child of a 30-year-old mother, and who at age 30 breast-fed her first and only child for 6 months. Nulliparous women were also assumed to have been breast-fed as the first and only child of a 30-year-old mother. Estimates to assess the influence of breast-feeding assumed that non–breast-fed children were fed formula derived from cow’s milk.

**Figure 1:**
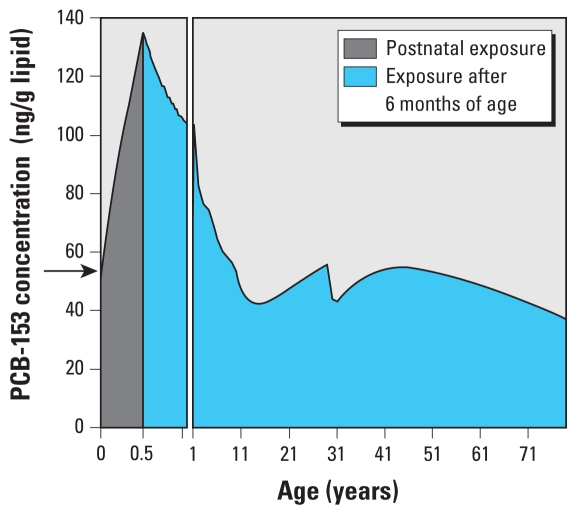
PCB-153 lipid-normalized concentration profile for a default female under constant emissions. Prenatal exposure (i.e., the estimated PCB-153 lipid-normalized concentration at birth) is indicated by the arrow. Postnatal exposure (gray area) is based on 6 months of breast-feeding. The entire area under the curve (gray plus blue areas) represents lifetime exposure.

A time step of 1 hr was used in all simulations with results plotted with a 5-day resolution for the first year of an individual’s life and on a 1-year resolution for years 1–80. Prenatal exposure was considered to be equal to the body burden at birth (Figure 1, arrow). Postnatal exposure was the cumulative exposure of the 6 months of lactational transfer. Lifetime exposure was defined as the cumulative exposure during the entire 80-year life span of a woman. Exposures are reported as lipid-normalized concentrations (nanograms per gram lipid), which should not be confused with overall body burden (nanograms per gram body weight or nanograms per person).

## Results and Discussion

### Human PCB exposure predictions under constant emissions

To interpret how lipid-normalized PCB concentrations are affected by time-variant emissions, it is helpful to understand the estimated concentration profile assuming constant emissions. Figure 1 illustrates the age dependence of the lipid-normalized PCB-153 concentration for a default female (as defined above). During the 6 months the woman is breast-fed as an infant, her estimated lipid-normalized PCB-153 concentration continually increases because the rate of PCB uptake from breast milk is assumed to be greater than the rate of growth (Figure 1). However, after breast-feeding ceases, the PCB concentration rapidly decreases because the rate of growth exceeds the rate of contaminant uptake from food until 12–15 years of age. Minor fluctuations in the concentration–time profile during this time period reflect the changing lipid content of the body (calculated according to Moser and McLachlan 2002) [see Supplemental Material, Figure 1 (doi:10.1289/ehp.1002415)]. By the time a woman reaches her late teens and early 20s her growth rate slows, and hence, with continued consumption of contaminated food, her lipid-normalized PCB-153 concentration increases (Figure 1). When a woman becomes pregnant, her body lipid content increases, resulting in a decrease in the overall lipid-normalized PCB-153 concentration (see Supplemental Material, Figure 1). Following childbirth at 30 years of age, the woman’s lipid-normalized PCB-153 concentration is further reduced by elimination of lipid-soluble contaminants through milk production (Figure 1). Upon weaning of her child, the mother’s predicted lipid-normalized PCB-153 concentration increases until it regains its prepregnancy concentration at approximately 40 years of age. We assume that after age 40 the relative proportion of lipid weight to body weight increases while the volume of food consumed decreases, resulting in declining PCB-153 concentrations (Figure 1). Several statistical studies suggest that the PCB body burden increases with age (Bergonzi et al. 2009; Dewailly et al. 1996). In fact, over the age ranges examined in these studies (16–42 years), our model also predicts increasing body burden with age. On the other hand, our prediction of decreased lipid concentration after 40 years of age is based on the assumption that body fat increases continually with age, which may not be true for all individuals.

### Human PCB exposure predictions under time-variant emissions

**Figure 2:**
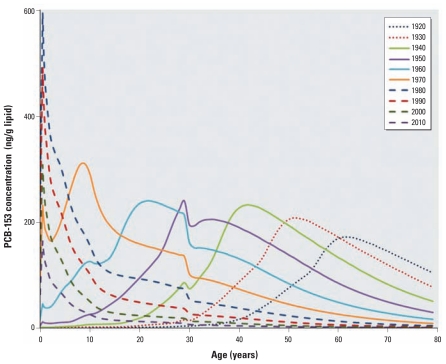
Predicted lifetime lipid-normalized concentration profiles of PCB-153 according to year of birth for default females born between 1920 and 2010 under time-variant conditions.

**Figure 3:**
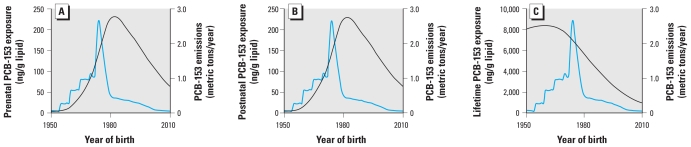
Comparison of prenatal (*A*), postnatal (*B*), and lifetime (*C*) lipid-normalized PCB-153 exposure predictions for default females according to year of birth (black line) based on estimated time-variant PCB-153 emissions (blue line). Note the difference in the *y*-axis scale in *C*.

Under nonsteady emissions, a default female’s lipid-normalized PCB-153 concentration profile depends on the age at which her exposure began and will reflect the variability in PCB emissions over time. This is evident in Figure 2, which shows estimated lipid-normalized PCB-153 concentrations predicted for default females born every 10 years between 1920 and 2010 under time-variant emissions [scenario 2; see Supplemental Material (doi:10.1289/ehp.1002415)]. Our model predicts that the largest peak concentration at any given age was experienced by 6-month-old girls born in 1980 (Figure 2), which suggests that maternal transfer during pregnancy and lactation was a significant source of PCB-153 contamination among women born after the PCB production phaseout in the 1970s (Figure 3). Although estimated PCB-153 concentrations peaked at 6 months of age in women born after 1980, peak concentrations for women born in 1970 or earlier would have occurred in the 1980s, regardless of a woman’s age during the 1980s (Figure 2). In other words, our model predicts that a woman born in 1940 would have experienced a peak PCB-153 concentration in 1980 at approximately 40 years of age, whereas a woman born in 1960 also would have experienced her peak concentration in 1980 at approximately 20 years of age. Although women born after maximum PCB-153 emissions would have experienced the highest maximum lipid-normalized PCB-153 concentrations, our model predicted that women born approximately 15 years before the phaseout (i.e., the generation born in 1960) would have experienced the greatest cumulative lifetime exposure to PCBs (Figure 3C). For model predictions for additional PCB congeners (28, 53, 101, 118, 138, 180), see Supplemental Material, Figure 3.

The predictions generated by our model are consistent with those reported by Alcock et al. (2000) and Moser and McLachlan (2002). In particular, the results of the present study and Alcock et al. (2000) suggest that the previously reported trend of increasing body burden with age was most likely an artifact of the year of birth relative to PCB emissions rather than increasing PCB exposure with age. The peak in dietary PCB contamination coincided with the peak in prenatal PCB exposure in the study by Moser and McLachlan (2002), whereas the results of our study predict that the peak in prenatal exposure occurred approximately 10 years after the maximum PCB emissions (Figure 3A). Therefore, this 10-year delay may represent the length of time required for PCBs to move from the air through the environment and into the food chain (Breivik et al. 2010). Simulations with different PCB emissions scenarios [see Supplemental Material, Figure 2 (doi:10.1289/ehp.1002415)] suggest that the temporal delay between generations with the highest cumulative lifetime PCB exposures and those with the highest prenatal and postnatal exposures was approximately 20–30 years regardless of the emissions scenario.

Our findings in this study imply that because the highest accumulated exposure occurred at reproductive age for women born in the 1960s (Figure 2), this group may be especially at risk for reproductive disturbances, as has been reported for both males and females with high organochlorine exposures (Toft et al. 2006). We estimated that the highest prenatal PCB-153 exposure occurred among children born in 1980 (Figure 3A), which suggests that this population may have been at increased risk of health effects associated with prenatal PCB exposure, including decreased motor skills, growth impairment, reduced IQ, reduced fecundity, and early-onset menstruation (Wigle et al. 2008). Therefore, even though women born in the 1960s may have experienced a greater cumulative exposure to PCBs than their children, their children may have suffered more health effects due to prenatal exposure. In other words, because of IGDE, the health repercussions of PCB exposures will span several generations but may manifest differently in each generation depending upon the timing of their exposure.

### Contributions of reproductive behaviors to IGDE under constant emissions

In addition to changing emission time trends, a secondary source of IGDE may arise from changing trends in reproductive behaviors. For example, in Sweden, the average age at first birth was 24 years in 1970, compared with 28.9 years in 2008, and the average number of children per woman was 2.1 in 1960 compared with 1.6 in 2000 (Statistics Sweden 2009). To differentiate reproductive trends from emissions variability, we first examined reproductive characteristics under a constant PCB emission scenario in which, once steady state has been reached, there would be no PCB IGDE assuming that reproductive behaviors are also held constant. In other words, under steady-state conditions, the mother and child would have the same concentration profile.

**Figure 4:**
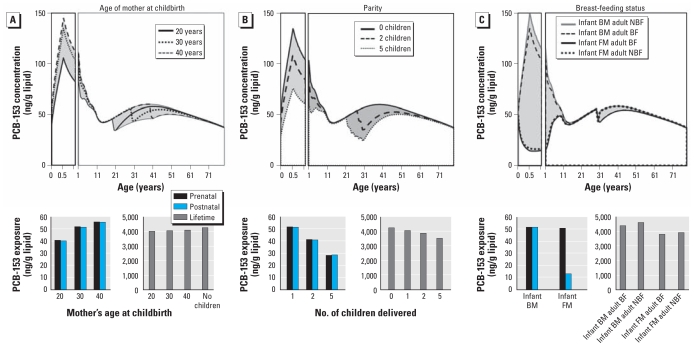
Predicted lipid-normalized PCB-153 profiles and estimated prenatal, postnatal, and lifetime exposures, assuming constant PCB-153 emissions over time for a woman who gives birth to her first and only child at 20, 30, or 40 years of age (*A*); has none, two, or five children (with births at 2-year intervals and the last birth at 30 years of age) (*B*); and consumed breast milk (BM) or formula milk (FM) exclusively for 6 months after her birth and breast-fed (BF) or formula-fed (NBF) a single infant born when the woman was 30 years of age (*C*). In a steady-state simulation, every generation experiences the same exposure (i.e., results for mother and child are the same).

Figure 4 shows comparisons of the predicted lifetime lipid-normalized PCB-153 concentration profiles of primiparous mothers at different ages and prenatal, postnatal, and cumulative lifetime exposure estimates under constant PCB emissions. The prenatal exposure of an infant born to a 20-year-old mother is 79% and 73% that of an infant born to a 30-year-old and 40-year-old mother, respectively, with similar differences in postnatal exposure according to the mother’s age at birth (Figure 4A). This supports the hypothesis that children born to mothers with greater lifetime PCB exposure will receive more maternal transfer (Bergonzi et al. 2009; Lignell et al. 2009; Uemura et al. 2008). However, the age at which the mother gives birth does not substantially influence the mother’s own estimated cumulative lifetime exposure (Figure 4A).

Figure 4B shows comparisons of the estimated lipid-normalized PCB-153 concentration profiles of a 30-year-old woman with five children (born at 2-year intervals at 22, 24, 26, 28, and 30 years of maternal age) and a nulliparous 30-year-old woman. Prenatal PCB-153 exposures of the second- and fifth-born children are predicted to be 80% and 55%, respectively, of the prenatal exposure of the first-born child, with similar predictions for postnatal exposure. Because we assumed that multiparous women breast-fed each child, the estimated decrease in both prenatal and postnatal exposure of the fifth-born child is a consequence of decreased body lipid burdens of PCB-153 in the mother due to transplacental transfer and breast-feeding of the previously born children. These results assume 6 months of breast-feeding and births at 2-year intervals; if breast-feeding continued > 6 months, PCB transfer from mother to child would increase and the prenatal and postnatal exposure of each successive child would decrease. On the other hand, prenatal and postnatal transfer to successive children would decrease as the amount of time between successive childbirths decreases because the body burden of the mother would have less time to recover to prepregnancy levels between births. A decrease in maternal transfer with increased parity might be expected to indicate large differences in the lifetime exposure of the mother (Schade and Heinzow 1998; Vaz et al. 1993; Wang et al. 2009). However, our model suggests that a mother who gives birth to one, two, or five children still experiences 96%, 91%, or 82%, respectively, of the PCB-153 lifetime exposure of a nulliparous woman (Figure 4B), supporting the hypothesis that parity has a minimal influence on the lifetime exposure of the mother (Bergonzi et al. 2009).

To differentiate the role of breast-feeding as a source of contamination during infancy and as a loss mechanism for the mother, we considered four breast-feeding scenarios (Figure 4C) depending on whether a woman was fed by breast milk or formula as an infant and whether she does or does not breast-feed one infant of her own. Alcock et al. (2000) estimated that the maternal PCB body burden would decrease by 25% during 6 months of breast-feeding, but our model only predicts a 5% decrease. The reason for the discrepancy may be that Alcock et al. assumed that 7.5 kg of a mother’s overall lipid content is transferred to a child during 6 months of breast-feeding, compared with only 4.5 kg assumed by our model. Furthermore, from the Alcock et al. (2000) study, it is unclear what the original lipid mass is for the mother who experiences a 25% decrease in body burden. The greater the proportion of lipid transferred to the infant, the greater the transfer of contaminant.

According to our model, at the age of 6 months, a formula-fed infant experiences 75% less postnatal PCB-153 exposure than does a breast-fed infant (Figure 4C). However, this translates into only a 15% reduction in lifetime exposure (Figure 4C). Furthermore, we estimate that breast-feeding as a loss mechanism accounts for < 5% of the difference in the lifetime exposure of a breast-feeding mother relative to a non–breast-feeding mother (Figure 4C). Of course, these predictions are based on 6 months of breast-feeding the infant 100% of the time. In reality, a mother may breast-feed her infant for ≥ 1 year, which would increase the amount of contaminant transferred to the infant, or may use a combination of breast milk and formula, which would decrease the transfer of contaminant.

From these constant emissions calculations, we can estimate the exposure variability within a population and between generations that can be quantitatively attributed to maternal age, parity, and breast-feeding prevalence. Our results suggest that parity and being breast-fed as an infant are the main factors contributing to variability in the lifetime PCB-153 exposure of the maternal population. On the other hand, breast-feeding an infant and age at childbirth do not significantly influence a woman’s own lifetime exposure. The previous number of offspring born to a mother is the greatest determinant of each infant’s prenatal exposure. Consequently, our findings suggest that a shift in the number of children per family over time will have a larger impact on intergenerational differences in both prenatal and lifetime PCB-153 exposures than will the other reproductive characteristics examined. Our findings also indicate that the type of milk fed to an infant has the greatest impact on postnatal exposure, and therefore the prevalence of breast-feeding also may have a strong influence on intergenerational differences in postnatal exposure. Under constant PCB-153 emissions, shifts in the age at which women give birth will also introduce intergenerational differences in both prenatal and postnatal exposures because as the average age of childbirth increases, prenatal and postnatal exposure will also increase (Bergonzi et al. 2009; Dewailly et al. 1996). However, changes in maternal age will have less of an impact on IGDE than will changes in the number of offspring and the prevalence of breast-feeding.

In terms of population impact, our results suggest that women born to older mothers and who have fewer older siblings may be the most likely to experience reduced fecundity and abnormal reproductive functioning due to their prenatal PCB-153 exposure. Interestingly, regardless of the reproductive characteristics of the mother, under constant emissions all offspring achieve equal lipid PCB-153 concentrations by 17 years of age (Ritter et al. 2009), and our model suggests that all women achieve approximately equal lipid PCB-153 concentrations by about 60 years of age (Figure 3A–C). This has potential implications for studies aiming to evaluate changing body burdens of a population over time. Specifically, researchers aiming to deduce trends in the PCB exposure experienced by a population over time should consider sampling females 17 years of age or > 60 years of age, because such sampling would reduce the variability caused by factors other than dietary contamination.

### Contributions of reproductive characteristics to bioaccumulation variability under time-variant emissions

**Figure 5:**
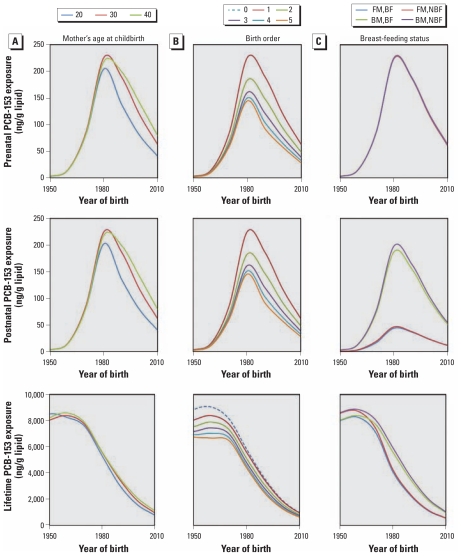
Predicted lipid-normalized prenatal, postnatal, and lifetime exposures to PCB-153 as a function of year of birth, assuming time-variant PCB-153 emissions for a woman who gives birth to her first and only child at 20, 30, or 40 years of age (*A*); has none, one, two, three, four, or five children (with childbirth at 2-year intervals and the fifth child born at 30 years of age for multiparous women) (*B*); and consumed breast milk (BM) or formula milk (FM) exclusively for 6 months after birth and breast-fed (BF) or formula fed (NBF) a single infant born when the woman was 30 years of age (*C*).

After evaluating the effects of time-variant PCB-153 emissions on IGDE and the impact of reproductive factors under constant PCB emissions on IGDE, we assessed the influence of reproductive characteristics in combination with emission variability, which is the scenario closest to reality. Figure 5 shows average estimated prenatal, postnatal, and lifetime PCB-153 exposures according to year of birth and reproductive characteristics. Our findings suggest that the age at which a mother gives birth influences only the prenatal and postnatal exposure of individuals born after the PCB production phaseout (Figure 5A) and that a younger mother (20 years of age) born after the phaseout would transfer less contaminant to an infant than would an older mother (30–40 years of age). With respect to lifetime exposure, the age of the mother at birth appears to introduce only minor variability in IGDE.

The extent of prenatal and postnatal exposure is greatest for the first-born child and less for each successive child, but again, this effect is observed only for women born after the PCB production phaseout (Figure 5B). On the other hand, the number of children that a woman has would affect her lifetime exposure only if her children were born before the PCB production phaseout. In this case, a nulliparous woman would have a higher lipid PCB-153 concentration than would a parous woman, and lifetime exposure would decrease with increasing parity. These results suggest that reproduction is a significant loss mechanism for PCBs, with respect to lifetime exposure, only for women born during the peak period of PCB use and production.

With respect to postnatal exposure, our findings suggest that the breast-fed child is exposed to more PCB contamination than is the formula-fed child regardless of year of birth, with the greatest contrast between breast-fed and formula-fed infants born in 1980 (Figure 5C). This suggests that breast milk is a significant source of contamination for infants. On the other hand, the difference in lifetime exposure is more complicated. We estimate that a woman who is breast-fed but does not breast-feed her own child will have the highest lifetime PCB exposure regardless of year of birth, whereas a woman who was formula fed as an infant but breast-feeds her own child will have the lowest lifetime exposure (Figure 5C). For women born between 1950 and 1980, differences between women who were breast-fed and also breast-feed a child and women who were formula-fed and do not breast-feed a child depend upon her year of birth relative to the PCB emissions scenario. In general, however, the overall difference between the four groups of women is relatively insignificant to their cumulative lifetime exposure.

Finally, as noted for the constant emissions scenario, by the time women reach 60 years of age, reproductive factors no longer contribute to population variability (for a population that is reproductively active only from 20 to 40 years of age). Furthermore, a mother’s reproductive characteristics appear to have a greater impact on the lipid PCB-153 concentration of her infant than on her own exposure, but the mother’s characteristics appear to be important only to infants born after the PCB production phaseout. This most likely reflects the amount of contaminant accumulated in the mother by the time she is of childbearing age. Most of the variation in the infant population can be attributed to feeding behavior, whereas the mother’s age at childbirth is relatively unimportant.

## Conclusion

We investigated the role of reproductive characteristics and time-variant emissions to improve our understanding of intergenerational differences in PCB lipid concentrations. Although our results suggest that women born before the PCB production phaseout experienced the highest cumulative lifetime exposure to PCBs, those born after the phaseout experienced higher prenatal and postnatal exposure. Because prenatal and postnatal PCB exposures have been associated with health complications, our results suggest that even though measures to phase out PCBs were initiated > 40 years ago, the health effects of PCBs are likely to persist over several generations.

## References

[b1-ehp-119-641] Alcock RE, Sweetman AJ, Juan CY, Jones KC (2000). A generic model of human lifetime exposure to persistent organic contaminants: development and application to PCB-101. Envion Pollut.

[b2-ehp-119-641] Aoki Y (2001). Polychlorinated biphenyls, polychloronated dibenzo-*p*-dioxins, and polychlorinated dibenzofurans as endocrine disrupters—what we have learned from Yusho disease. Environ Res.

[b3-ehp-119-641] Bergonzi R, Specchia C, Dinolfo M, Tomasi C, De Palma G, Frusca T (2009). Distribution of persistent organochlorine pollutants in maternal and foetal tissues: data from an Italian polluted urban area. Chemosphere.

[b4-ehp-119-641] Breivik K, Czub G, McLachlan MS, Wania F (2010). Towards an understanding of the link between environmental emissions and human body burdens of PCBs using CoZMoMAN. Environ Int.

[b5-ehp-119-641] Chen PH, Gaw JM, Wong CK, Chen CJ (1980). Levels and gas chromatographic patterns of polychlorinated biphenyls in the blood of patients after PCB poisoning in Taiwan. Bull Environ Contam Toxicol.

[b6-ehp-119-641] Czub G, McLachlan MS (2004). A food chain model to predict the levels of lipophilic organic contaminants in humans. Environ Toxicol Chem.

[b7-ehp-119-641] Dewailly É, Ayotte P, Laliberté C, Weber JP, Gingras S, Nantel AJ (1996). Polychlorinated biphenyl (PCB) and dichlorodiphenyl dichloroethylene (DDE) concentrations in the breast milk of women in Quebec. Am J Public Health.

[b8-ehp-119-641] Fujiwara K (1975). Environmental and food contamination with PCB’s in Japan. Sci Total Environ.

[b9-ehp-119-641] Guo YL, Hsu PC, Hsu CC, Lambert GH (2000). Semen quality after prenatal exposure to polychlorinated biphenyls and dibenzofurans. Lancet.

[b10-ehp-119-641] Hsu PC, Huang W, Yao WJ, Wu MH, Guo YL, Lambert GH (2003). Sperm changes in men exposed to polychlorinated biphenyls and dibenzofurans. JAMA.

[b11-ehp-119-641] Jacobson JL, Jacobson SW, Humphrey HEB (1990). Effects of in utero exposure to polychlorinated biphenyls and related contaminants on cognitive functioning in young children. J Pediatr.

[b12-ehp-119-641] Lignell S, Aune M, Darnerud PO, Cnattingius S, Glynn A (2009). Persistent organochlorine and organobromine compounds in mother’s milk from Sweden 1996–2006: compound specific temporal trends. Environ Res.

[b13-ehp-119-641] Moser GA, McLachlan MS (2002). Modeling digestive tract absorption and desorption of lipophilic organic contaminants in humans. Environ Sci Technol.

[b14-ehp-119-641] Ritter R, Scheringer M, MacLeod M, Schenker U, Hungerbuhler K (2009). A multi-individual pharmacokinetic model framework for interpreting time trends of persistent chemicals in human populations: application to a postban situation. Environ Health Perspect.

[b15-ehp-119-641] Safe SH (1994). Polychlorinated biphenyls (PCBs): environmental impact, biochemical and toxic responses, and implications for risk assessment. Crit Rev Toxicol.

[b16-ehp-119-641] Schade G, Heinzow B (1998). Organochlorine pesticides and polychlorinated biphenyls in human milk of mothers living in northern Germany: current extent of contamination, time trend from 1986 to 1997 and factors that influence the levels of contamination. Sci Total Environ.

[b17-ehp-119-641] Statistics Sweden (2009). Summary of Population Statistics 1960–2008 (corrected version 2009–05–13).

[b18-ehp-119-641] Tajimi M, Watanabe M, Oki I, Ojima T, Nakamura Y (2004). PCDDs, PCDFs and Co-PCBs in human breast milk samples collected in Tokyo, Japan. Acta Paediatr.

[b19-ehp-119-641] Toft G, Hagmar L, Giwercman A, Bonde JP (2004). Epidemiological evidence on reproductive effects of persistent organochlorines in humans. Reprod Toxicol.

[b20-ehp-119-641] Toft G, Rignell-Hydbom A, Tyrkiel E, Shvets M, Giwercman A, Lindh CH (2006). Semen quality and exposure to persistent organochlorine pollutants. Epidemiology.

[b21-ehp-119-641] Uemura H, Arisawa K, Hiyoshi M, Satoh H, Sumiyoshi Y, Morinaga K (2008). PCDDs/PCDFs and dioxin-like PCBs: recent body burden levels and their determinants among general inhabitants in Japan. Chemosphere.

[b22-ehp-119-641] Vaz R, Slorach SA, Hofvander Y (1993). Organochlorine contaminants in Swedish human milk: studies conducted at the National Food Administration 1981–1990. Food Addit Contam.

[b23-ehp-119-641] Wang RY, Jain RB, Wolkin AF, Rubbin CH, Needham LL (2009). Serum concentrations of selected persistent organic pollutants in a sample of pregnant females and changes in their concentrations during gestation. Environ Health Perspect.

[b24-ehp-119-641] Wania F, Breivik K, Persson NJ, McLachlan MS (2006). CoZMo-POP 2—a fugacity-based dynamic multi-compartmental mass balance model of the fate of persistent organic pollutants. Environ Modell Softw.

[b25-ehp-119-641] Wigle DT, Arbuckle TE, Turner MC, Bérubé A, Yang QY, Liu SL (2008). Epidemiologic evidence of relationships between reproductive and child health outcomes and environmental chemical contaminants. J Toxicol Environ Health B Crit Rev.

